# An evaluation of two-channel ChIP-on-chip and DNA methylation microarray normalization strategies

**DOI:** 10.1186/1471-2164-13-42

**Published:** 2012-01-25

**Authors:** Michiel E Adriaens, Magali Jaillard, Lars MT Eijssen, Claus-Dieter Mayer, Chris TA Evelo

**Affiliations:** 1 Department of Bioinformatics - BiGCaT, Maastricht University, Maastricht, The Netherlands; 2 Netherlands Consortium for Systems Biology (NCSB), University of Amsterdam, The Netherlands; 3 Department of Biomathematics and Statistics Scotland, University of Aberdeen, Rowett Institute of Nutrition and Health, Aberdeen, UK

## Abstract

**Background:**

The combination of chromatin immunoprecipitation with two-channel microarray technology enables genome-wide mapping of binding sites of DNA-interacting proteins (ChIP-on-chip) or sites with methylated CpG di-nucleotides (DNA methylation microarray). These powerful tools are the gateway to understanding gene transcription regulation. Since the goals of such studies, the sample preparation procedures, the microarray content and study design are all different from transcriptomics microarrays, the data pre-processing strategies traditionally applied to transcriptomics microarrays may not be appropriate. Particularly, the main challenge of the normalization of "regulation microarrays" is (i) to make the data of individual microarrays quantitatively comparable and (ii) to keep the signals of the enriched probes, representing DNA sequences from the precipitate, as distinguishable as possible from the signals of the un-enriched probes, representing DNA sequences largely absent from the precipitate.

**Results:**

We compare several widely used normalization approaches (VSN, LOWESS, quantile, T-quantile, Tukey's biweight scaling, Peng's method) applied to a selection of regulation microarray datasets, ranging from DNA methylation to transcription factor binding and histone modification studies. Through comparison of the data distributions of control probes and gene promoter probes before and after normalization, and assessment of the power to identify known enriched genomic regions after normalization, we demonstrate that there are clear differences in performance between normalization procedures.

**Conclusion:**

T-quantile normalization applied separately on the channels and Tukey's biweight scaling outperform other methods in terms of the conservation of enriched and un-enriched signal separation, as well as in identification of genomic regions known to be enriched. T-quantile normalization is preferable as it additionally improves comparability between microarrays. In contrast, popular normalization approaches like quantile, LOWESS, Peng's method and VSN normalization alter the data distributions of regulation microarrays to such an extent that using these approaches will impact the reliability of the downstream analysis substantially.

## Background

For over a decade, two-channel transcriptomics microarrays have provided a powerful approach to study genome-wide gene expression events. Now, continued development of two-channel microarray technology has enabled extending our experimentation to the next level: regulation of gene transcription. One of the most popular techniques in this field combines chromatin immunoprecipitation (ChIP) assays with two-channel microarray technology (ChIP-on-chip [1]). ChIP-on-chip studies are used to detect any protein-DNA interaction genome-wide, such as transcription factor binding, but also epigenetic events such as histone modifications, as long as a suitable antibody is available. The same approach is used to detect DNA methylation, by using either an antibody that interacts with methyl-CpG-binding domain (MBD) proteins bound to methylated CpG dinucleotides (MBD-ChIP assay), or an antibody that interacts with methylated CpG dinucleotides directly (methylated DNA immunoprecipitation (MeDIP) assay [2]).

Even though newer technologies such as ChIP-sequencing (ChIP-seq) are on the rise, two-channel microarrays still present a valuable approach to understanding gene transcription regulation events, and during the last decade have opened opportunities to identify novel targets and markers in complex diseases such as cancer [3-5], heart failure [6] and diet-related disorders [7], and psychiatric disorders such as depression, schizophrenia and addiction [8]. Since the main appliance of this technology at the time being is gene transcription regulation studies - transcription factor and co-regulator binding, DNA methylation, and histone modifications - the term 'regulation microarrays' will be used for brevity henceforth.

The design and the experimental approach for regulation microarrays are very different from the more extensively studied transcriptomics microarrays, which has implications for data pre-processing procedures. The key difference is that in transcriptomics microarrays both channels contain amplified transcript samples, usually corresponding to two different experimental conditions, while in regulation microarrays the channels comprise an experimental sample and a reference sample. The cyanine 3 (Cy3), or green, channel of regulation microarrays generally contains the total DNA sample that gives the reference baseline signal, and the cyanine 5 (Cy5), or red, channel contains an experimentally enriched DNA sample, extracted using a specific antibody binding to a DNA-interacting protein (ChIP) or directly to methylated CpGs on the DNA (MeDIP). Hence, while the log-ratio between the channel signals represents the differential expression between two conditions in transcriptomics studies, for regulation microarrays it is used as a measure of enrichment: the higher the log-ratio of a probe or set of tiling probes, the higher the likelihood that the corresponding region in the genome has a high level of methylation or is targeted by a DNA-interacting protein.

**Figure 1:**
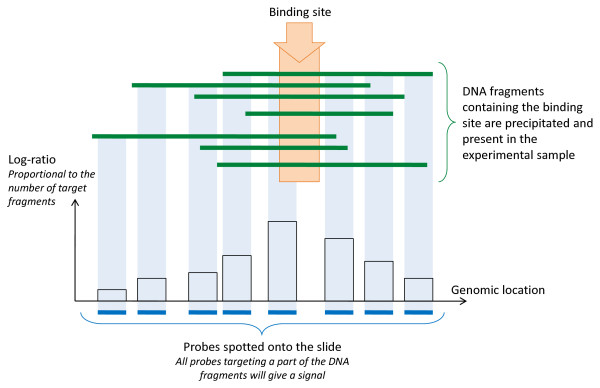
**The birth of an enrichment signal around a binding site (ChIP-on-chip)**. Since DNA fragmentation through sonication can be modeled as a Poisson process [1], the DNA fragment length distribution follows a Poisson distribution and adjacent probes on the genome have a correlated log-ratio, resulting in the hybridization pattern shown here. Each blue column represents a probe hybridization site. Black-outlined bars represent their log-ratio. Green lines are sonicated immuno-precipitated DNA fragments corresponding to the binding site.

**Figure 2:**
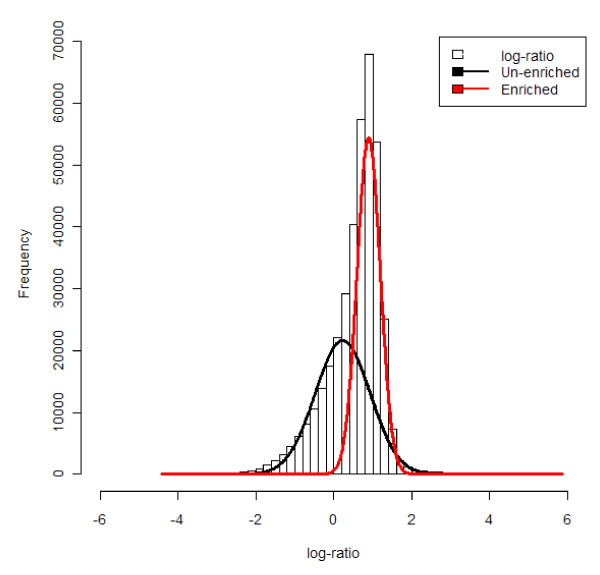
**An example of a two-component distribution fitted on ChIP-on-chip data of dataset #1 (see Methods section for dataset description and numbering)**.

Another important assumption in regulation microarrays is that a DNA-interacting protein is either bound or not bound (for ChIP) and that a target sequence is either methylated or not (for MeDIP). Regardless, depending on binding affinity, mean time of residence and other factors, the fraction of cells with bound protein or a particular methylation status is not an all-or-nothing condition, especially in heterogeneous tissues. Combined with the characteristics of the data distribution surrounding a site of interest (Figure 1) and probe effects [9], this produces a continuous log-ratio distribution. However, the characteristics of the samples hybridized to the channels force a dichotomy upon the log-ratio distribution, which is comprised of two components (Figure 2) commonly referred to as an enriched and an un-enriched component [10]. The enriched component corresponds to the probes to which the experimental DNA has hybridized and the un-enriched component to the probes whose targets are largely absent from the experimental DNA sample. Hence, contrary to transcriptomics microarray data, where low log-ratio values are meaningful as long as the differences between conditions are statistically significant, when interpreting ChIP-on-chip and DNA methylation microarray data, the upper quantile is of most interest, as it generally comprises mostly enriched probes. Based on this assumption, enrichment finding algorithms like ACME [11], will test if a set of tiling probes is significantly more likely to be a sampling of this upper quantile than of the rest of the data, assuming that if this is the case, this set of tiling probes corresponds to a protein binding site or methylated region. A better separation between the enriched and un-enriched components hence increases the power to identify enriched regions. Thus, a crucial aspect in regulation studies is that any separation between the enriched and un-enriched components present in the data before normalization, should be kept afterwards. Apart from conserving this separation, other aspects need to be taken into account when normalizing regulation microarray data.

Normalization is a process that is applied at multiple levels connected to spatial [12], probe [13-15] and dye or intensity dependent biases [16]. Additionally, differences in print quality, differences in ambient conditions when the plates were processed or changes in the scanner settings can cause scaling differences between microarrays. Most of the assumptions underlying the process of correcting for these biases are identical for transcriptomics microarrays and regulation microarrays. The exception is the correction for intensity dependent bias, for which the most common approaches in use for transcriptomics microarrays are LOWESS normalization [12,17,18] and quantile normalization [19]. Both methods are based on the assumption that the majority of probe signals are unchanged between channels and microarrays, which generally holds for transcriptomics studies [12,20,18]. In regulation studies however, this assumption does not hold since the samples comprising the two channels differ to a large extent.

Based on these observations, the main challenge of the normalization of regulation microarrays is (i) to make the signals of individual microarrays quantitatively comparable and (ii) to retain the separation between the enriched and un-enriched components present in the data. Programs like CoCAS [21] offer a range of normalization methods for regulation microarrays, including quantile normalization [19] and variance stabilizing normalization [22], and R/Bioconductor [23] offers many more popular choices, which may not all be suitable for this challenge. Hence, we here assess the efficacy in removing technical biases and in preservation of the separation between the enriched and un-enriched components, for six two-channel microarray normalization methods (VSN [22], LOWESS [12,16], quantile [19], T-quantile [19], Tukey's biweight scaling, Peng's method [24]) applied to five published ChIP-on-chip and MeDIP-on-chip datasets on the NimbleGen platform.

## Results

To determine the efficacy in correcting for technical biases and improving comparability between microarrays, quality control and bias assessment was performed on all datasets before and after normalization for each of the six normalization approaches. Complete results are available in additional file 1 and 2. In all datasets scaling effects between microarrays and intensity dependent bias within microarrays are present, visible from the microarray data distributions. All tested normalization methods are able to correct for the observed biases, where from a technical standpoint, normalization approaches that normalize channels together (VSN, LOWESS, Peng's method, quantile) equalize the data distributions to a larger extent than normalization approaches that normalize the channels separately (T-quantile, Tukey's biweight scaling). In the latter category, T-quantile normalization enhances overall comparability to a larger extent than Tukey's biweight scaling.

**Figure 3:**
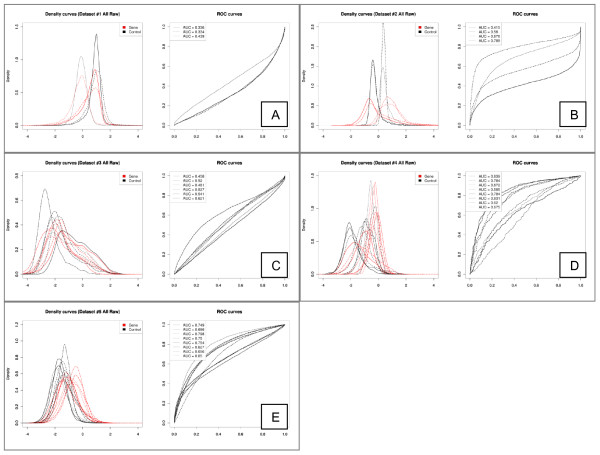
**Density distributions of the control probes and gene promoter probes of the raw log-ratio data of all individual microarrays and corresponding ROC curves for dataset #1 (a), dataset #2 (b), dataset #3 (c), dataset #4 (d) and dataset #5 (e)**. AUC values of each ROC curve are reported in the legend.

To evaluate the separation between the enriched and un-enriched components, the gene promoter probe and the negative control probe log-ratio distributions were assessed using ROC curves before and after normalization with each of the six normalization approaches (Figure 3). The raw data from dataset #1 (see **Methods **section for dataset details and numbering) shows largely overlapping control probe and gene promoter probe distributions (Figure 3a). Between individual microarrays, the distributions show larger differences, also resulting in more variation in both the area under the curve (AUC) as well as the shape of the ROC curves, indicating that comparability between microarrays is hindered by lack of normalization.

**Figure 4:**
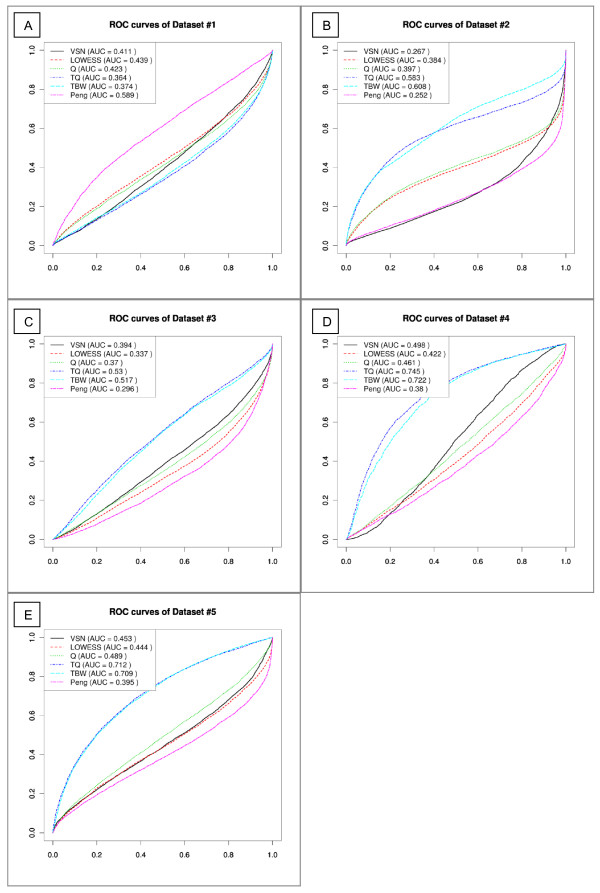
**ROC curves of the control probe and gene promoter distributions of the combined log-ratio data, for each normalization approach of dataset #1 (a), dataset #2 (b), dataset #3 (c), dataset #4 (d) and dataset #5 (e)**. AUC values are reported in the legend. TBW = Tukey's biweight scaling, Q = quantile normalization, TQ = T-quantile normalization.

The results of the combined data of the individual microarrays from the six normalization approaches (VSN, LOWESS, quantile, T-quantile, Tukey's biweight scaling, Peng's method) show equal performance of all approaches for dataset #1 (Figure 4a), resulting in ROC curves with similar shape and comparable AUC values. Based on the AUC values, separation between components is best when using Peng's method.

**Figure 5:**
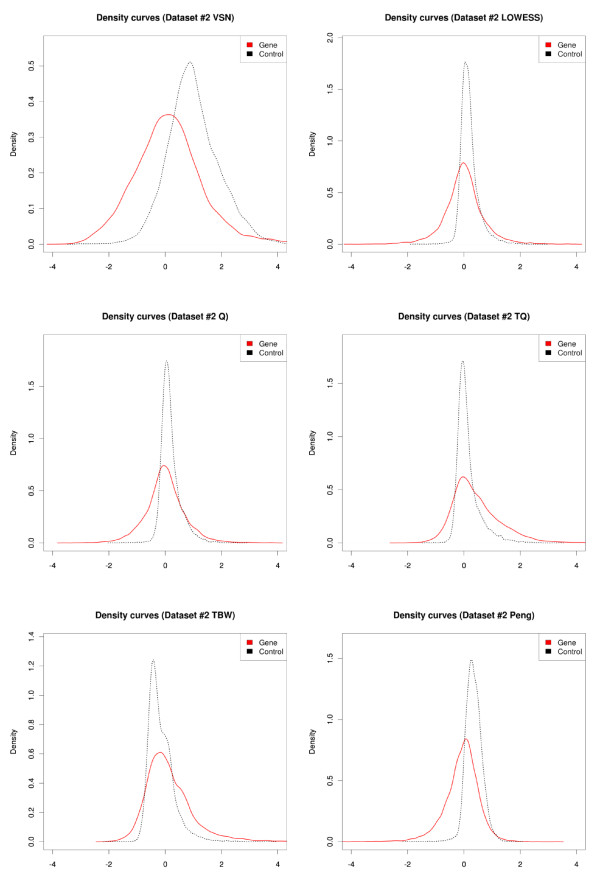
**Density distributions of the control probes and gene promoter probes of the normalized combined log-ratio data of dataset #2 (ChIP-on-chip)**. Results are shows for (from left to right, top to bottom) VSN, LOWESS, quantile (Q), T-quantile (TQ), Tukey's biweight scaling (TBW), Peng's method.

Dataset #2, the second ChIP-on-chip dataset gives different results (Figure 3b and 4b). Separation between components is preserved best when using T-quantile or Tukey's biweight scaling normalization (Figure 4b). The other approaches, including Peng's method, alter the ranking of probes resulting in the control probe and gene promoter probe distributions becoming superimposed. VSN normalization appears to scale the distributions, enforcing a larger spread compared to the data acquired through the other normalization approaches (Figure 5).

**Figure 6:**
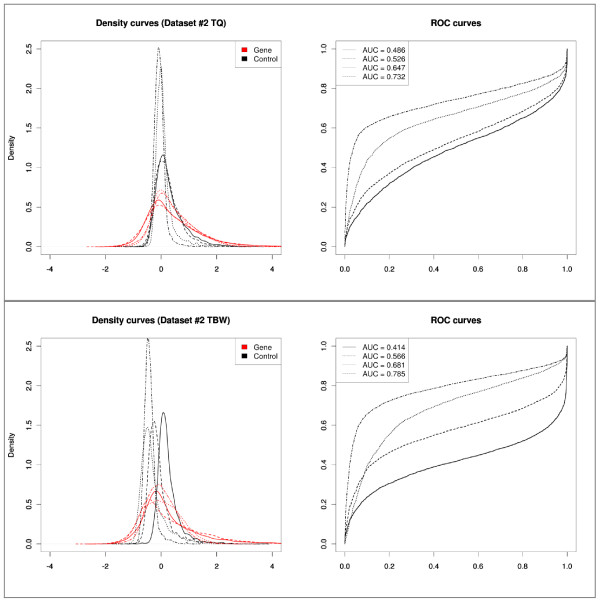
**Density distributions of the control probes and gene promoter probes of the normalized log-ratio data of each individual microarray and corresponding ROC curves of dataset #2 (ChIP-on-chip)**. Top: Results for T-quantile (TQ) normalized data. Bottom: Results for Tukey's biweight scaling (TBW) normalized data. AUC values of each ROC curve are reported in the legend.

Tukey's biweight scaling and T-quantile normalization appear to perform comparably with respect to conserving the component separation. Tukey's biweight scaling adjusts the log-ratio data with a scaling factor for each microarray in the dataset individually, which means that the ROC curves will be identical to those of the raw data, and that the distributions will be the same as those before normalization save for a shift. This may explain the variability observed in the individual ROC curves and AUC values of the Tukey's biweight scaling normalized data. T-quantile normalization reduces the variability between the data distributions of individual microarrays, resulting in ROC curves that are more comparable in both shape and AUC (Figure 6).

**Figure 7:**
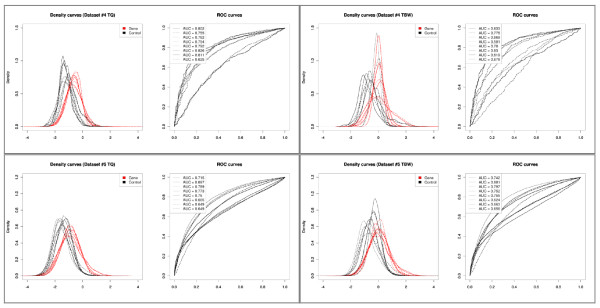
**Density distributions for the control probes and gene promoter probes of the normalized log-ratio data of each individual microarray and corresponding ROC curves of dataset #4 and #5**. Top left: Results for T-quantile (TQ) normalized data of dataset #4. Top right: Results for Tukey's biweight scaling (TBW) normalized data of dataset #4. Bottom left: Results for T-quantile (TQ) normalized data of dataset #5. Bottom right: Results for Tukey's biweight scaling (TBW) normalized data of dataset #5. AUC values of each ROC curve are reported in the legend.

The results of the MeDIP-on-chip datasets support the conclusions reached for the ChIP-on-chip datasets: separation of the components present before normalization (Figure 3c, d and 3e) are preserved best with T-quantile and Tukey's biweight scaling approaches (Figure 4c). LOWESS, quantile, VSN and Peng's normalization alter the distributions and eradicate the separation. In dataset #3, the differences between the normalization approaches is less striking, illustrated by similarly shaped ROC curves and AUC values (Figure 4c). Dataset #4 shows a larger heterogeneity between individual microarrays than both dataset #3 and #5. For both dataset #4 and #5 Tukey's biweight scaling and T-quantile normalization produce higher AUC values for these approaches (Figure 4d and 4e). Both methods appear to perform comparably with respect to conserving the component separation. However, as in dataset #2, the differences between both approaches are highlighted by the distributions of the individual microarrays: Tukey's biweight scaling adjusts each microarray individually, whereas T-quantile normalization is applied between microarrays. T-quantile normalization thereby results in ROC curves with less variation in shape and AUC (Figure 7) than those of the raw data and the Tukey's biweight scaling normalized data.

Any appropriate normalization method should preserve the biological information present in the raw data. Assessing the distributions of the negative control probes and the gene promoter probes is a global indicator of this conservation of biological information. In addition, three datasets with suitable positive controls were used to assess the impact of the normalization approaches on the power to identify significant enrichment for specific genomic regions. ACME [11] was used for all enrichment calculations. For dataset #1, 33 validated ER-a targets were used as positive controls [25,26]. The results are reported in table 1 for all normalization approaches and for several enrichment p-value cut-offs (0.05, 0.10, 0.20 and 0.50). T-quantile and quantile normalization in general result in identification of more targets at each cut-off.

**Table 1:** Number of validated estrogen receptor α targets found significantly enriched in the estrogen receptor α ChIP-on-chip dataset (dataset #1).

	Number of ER-a targets found (out of 33)			
**Normalization approach**	**Enrichment p-value** **< 0.05**	**< 0.10**	**< 0.20**	**< 0.50**

VSN	7	8	11	23

LOWESS	6	7	10	25

Quantile	8	9	12	25

T-quantile	9	10	14	24

Tukey's biweight scaling	7	8	11	23

Peng's method	5	5	9	23

Table 1 contains for each of the tested normalization approaches the number of validated estrogen receptor α targets [25] found significantly enriched in the estrogen receptor α ChIP-on-chip dataset (dataset #1). Results for four enrichment p-value cut-offs are given (0.05, 0.10, 0.20 and 0.50).

**Figure 8:**
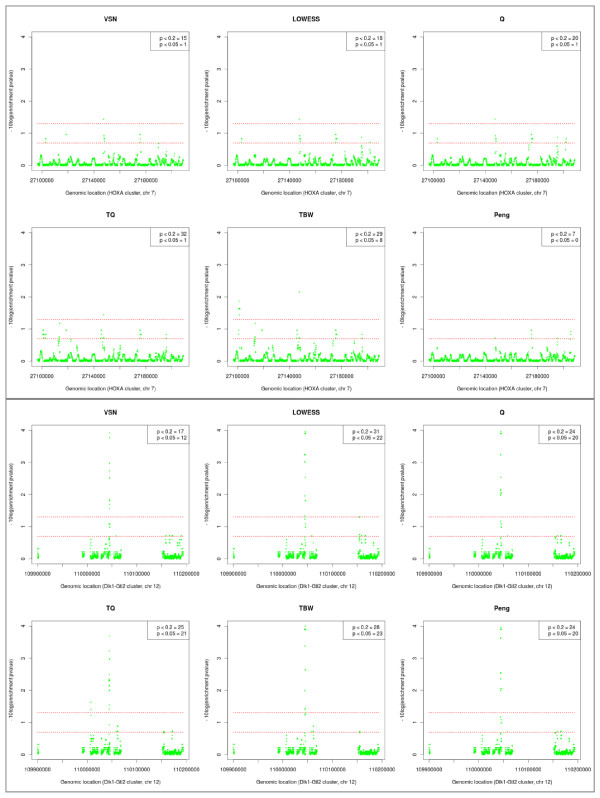
**Genome plots of negative ^10^log-transformed enrichment p-values, for the HOXA cluster on human chromosome 7 (top) and the Dlk1-Gtl2 cluster on mouse chromosome 12 (bottom)**. Red vertical lines are given at values corresponding to p-values of 0.05 (top line) and 0.20 (bottom line). Regions with values above the top line are highly enriched, while values between the lines are a sign of moderate enrichment. The total number of identified enriched regions are reported in the legend. TBW = Tukey's biweight scaling, Q = quantile normalization, TQ = T-quantile normalization.

For dataset #3 enrichment of the HOXA group of developmental genes was calculated. HOXA genes are located in a cluster on chromosome 7 and are known to be switched off and moderately to highly methylated in most tissues [27]. The negative ^10^log-transformed enrichment p-values plotted along the HOXA region are shown in Figure 8 (**top**). Using Tukey's biweight scaling or T-quantile normalization results in identification of several enriched loci, most of which are moderately methylated. Less loci are found when using VSN, quantile or LOWESS normalization. Peng's method results in identification of only a few loci with moderate enrichment.

For dataset #4 enrichment was determined for the Dlk1-Gtl2 cluster on chromosome 12, a region reported in the original results [28] to be highly enriched. For all normalization approaches in dataset #4 the same area in this region is identified as very highly enriched (Figure 8**bottom**).

## Discussion

Two-channel transcriptomics and regulation microarrays should not be pre-processed in the same manner. Appropriate normalization strategies for regulation microarrays are characterized by their ability to retain the separation between the enriched and un-enriched components present in the data whilst enhancing comparability between microarrays. Six normalization methods were tested by (i) assessing the separation between the control probe and gene promoter probe distributions before and after normalization using ROC curves and (ii) by verifying whether known enriched genes and regions could be identified as such after normalization. We have shown that the result of each approach depends heavily on the situation before normalization, specifically the amount of enriched and un-enriched probes and the separation between the corresponding components in the raw data. These two characteristics are different for each experiment, depending largely on the biological system studied and the applied assay.

In the ChIP-on-chip datasets used here, the distributions of the control probes and gene promoter probes overlap to a large extent before and after normalization. This may be explained by the small proportion of the genome generally covered by the potential binding sites of a DNA-interacting protein and the resulting small contribution of the enriched component. Hence in general, the lower the amount of binding sites, the more similar the control probe and gene promoter distributions, and the more comparable the performance of the normalization approaches, based on ROC curves of both distributions before and after normalization. However, in some cases VSN can cause a sizeable rescaling of the distributions, and to a spurious control probe distribution with a higher mean and spread than the gene promoter probe distribution. This renders gene promoter probes in the upper quantile of the data indistinguishable from random data, strongly impacting the biological interpretation.

In DNA methylation microarrays the amount of enriched probes and un-enriched probes is of the same order, since in general the proportion of methylated CpG di-nucleotides in a genome is substantial. We have shown that for such microarrays, the choice for a normalization procedure will be crucial for the downstream analysis. All three MeDIP-on-chip datasets show a large degree of separation between the gene promoter and control probe distributions. The separation is lost when using normalization methods that normalize channels together, such as VSN, LOWESS, Peng's method and quantile normalization. Using LOWESS approaches on MeDIP-on-chip data has been reported elsewhere to result in increased bias, because the underlying assumption that the log-ratio should be independent of the average individual channel signals does not hold for this type of data. DNA methylation levels are related to CpG and GC density, while signal intensity is also known to be influenced by GC content. Forcing the log-ratio to be independent of the average signal intensity using LOWESS normalization thus introduces bias instead of removing it [9].

T-quantile normalization, applied separately on the channels, and Tukey's biweight scaling are the only approaches that are able to preserve the component separation in all example datasets. In dataset #1, individual microarrays already showed comparable distributions before normalization; hence for this dataset, Tukey's biweight scaling would be sufficient. In contrast, dataset #4 for example showed a large heterogeneity between individual microarrays, in which case between-microarray normalization is better suited to improve the overall comparability and enable quantitative data comparison. This can be achieved either by doing an additional normalization step after scaling, but ideally by using a between-microarray normalization approach from the beginning, such as applying T-quantile normalization as demonstrated here.

In regulation microarrays the sequence content of the input DNA sample and the experimental DNA sample always differs to a large extent. There are also instances for transcriptomics microarrays, such as dedicated microarrays designed for a specific biological context, where the assumption that the majority of genes are not differentially expressed does not hold, hence requiring adapted normalization strategies. Most of these strategies involve the use of invariant genes, either present on the slide [29,30] or determined from the data [31]. Selecting invariant probes in ChIP-on-chip and DNA methylation data is difficult however, even when selecting the control probes used in the analysis presented here, because this would implicate a normalization based on un-enriched probes. Since the sequences meant to hybridize to these probes are largely absent from the experimental sample, they essentially measure background noise in the channel containing the experimental sample. Variation in log-ratio values of these un-enriched probes between microarrays therefore reflects methodological effects rather than biology, which compromises their usability. To avoid the use of invariant genes in transcriptomics microarrays, a three-component mixture model has been proposed [32]. The normalization parameters are estimated independently in the groups of up-regulated, down-regulated and unchanged genes and normalized separately. Such a model in adapted form can be fitted on regulation microarray data and used conjointly with enrichment finding. It has been shown that for DNA methylation studies using specific reference samples, such as a fully methylated total DNA sample, it is possible to make robust estimates for methylation percentages when using such a model [9,33,34].

The research described herein is limited to the normalization of replicate microarrays. In many cases however, a study will consist of multiple conditions, such as different tissues, or treatment and control samples as demonstrated in dataset #1. In these cases, the experimental DNA samples may differ to a large extent between treatment and control groups, warranting application of normalization to each condition separately. However, when only a relatively small amount of loci is expected to be differentially enriched and the total amount of enrichment can be assumed constant between conditions, normalization approaches applied to the dataset as a whole are more appropriate. This holds for experiments such as DNA methylation studies on the same tissue treated with a micronutrient [35], where only a projected limited amount of important regulatory regions with substantially altered levels of methylation is of interest.

The results of known targets and enriched regions show consistent differences between the various normalization approaches. When looking at the Dlk1-Gtl2 cluster for the DNA methylation data of dataset #4, a region reported to be highly enriched in the original findings, it is clear that such highly enriched regions will be identified as such regardless of the chosen normalization approach. This is not the case when studying moderately enriched regions, as illustrated by the results of the HOXA cluster in dataset #3, where the degree to which this region is identified as being enriched depends strongly on the applied normalization approach. Overall, T-quantile normalization and Tukey's biweight scaling again give the best results. A potential cause of the observed difference between the tested normalization approaches is observed in the results on global level: the ranking of probes changes when using some normalization approaches, increasing the likelihood of un-enriched probes being spread over the whole dynamic range of the enriched probe distribution. Ultimately, such changes in the ranking can be destructive on the power to call differences in methylation or protein binding. Also, enrichment finding algorithms [11] as used for these results, will test if a group of tiling probes is significantly more likely to be part of the upper quantile than of the rest of the data distribution, assuming that if this is the case, this group of tiling probes shows significant enrichment and thus corresponds to a binding site or methylated region. This upper quantile can be defined for each microarray individually after normalization. Hence, it is not the values themselves, but the rank in the data distribution which is biologically relevant. Considering this, within channel and treatment normalization approaches do not only enable a more robust data interpretation, but since for many applications the individual values themselves do not need to be comparable, they are also sufficient.

## Conclusion

The main issue of ChIP-on-chip and DNA methylation microarray normalization is to enhance comparability between microarrays, while keeping the separation between the enriched and un-enriched components present in the data. Within-channel approaches give the best performance, with enhanced comparability between individual microarrays for approaches that also normalize between microarrays. More specifically, quantile, LOWESS, Peng's and VSN normalization alter the signal distributions to such an extent that it will impact the reliability of the downstream analysis substantially. Better results are obtained with T-quantile normalization applied separately on the channels or Tukey's biweight scaling. For all datasets tested, these two methods consistently outperform the other tested methods in conservation of separation between the enriched and un-enriched distributions, as well as in identification of genomic regions known to be enriched. The T-quantile approach is preferable because it additionally yields enhanced comparability between microarrays.

## Methods

### ChIP-on-chip and DNA methylation microarray dataset selection

Five published datasets were selected from ArrayExpress. Selection criteria were set to select several assay types (MeDIP and ChIP), several species (human and mouse) and cover several research fields. Due to the selection criteria, all datasets were chosen from the same microarray manufacturer, NimbleGen (table 2).

**Table 2:** Technical information on the ChIP-on-chip and MeDIP-on-chip datasets used for the normalization approach comparison.

Dataset number	#1	#2	#3	#4	#5
**ArrayExpress ID**	not registered	E-TABM-529	E-GEOD-17581	E-GEOD-24286	E-GEOD-22831

**Assay type**	ChIP-on-chip	ChIP-on-chip	MeDIP-on-chip	MeDIP-on-chip	MeDIP-on-chip

**Microarray ID**	NimbleGen Human HGS17 minimal promoter	NimbleGen Mouse Tiling 2006-07-17 MM8Tiling Set17	NimbleGen Homo sapiens 385 K CGH array	NimbleGen mouse 385 K Refseq and miRNA promoter tiling (2-array set)	NimbleGen Nimblegen HD2 MM8 promoter deluxe array

**Species**	Human	Mouse	Human	Mouse	Mouse

**Investigation**	Identification of ER-α target genes in breast cancer cells	Identification of histone modification profiles in WT and Kcnq1ot1	Methylome analysis of congenital ectopic thyroids	Mecp2-dependent regulation of MicroRNAs in Rett Syndrome	DNA methylation analysis in E3.5 blastocysts, E6.5 epiblasts and E9.5 whole embryos

**No of microarrays**	8	11	6	8	11

**Microarray content**	3 stimulated by 17beta-estradiol 1 pool of the 3 stimulated 3 untreated 1 pool of the 3 untreated	2 Kcnq1ot1 9 wild type Tissues are placenta or liver	3 orthotopic thyroid 3 congenital ectopic thyroid	2 KO using Mecp2 4 wild type using Mecp2 2 wild type using 5-methylcytosine	2 E3.5 blastocysts 3 E6.5 epiblasts 3 E9.5 whole embryos 3 Control pooled unamplified MeDIPs in E9.5 embryos

**Microarrays used for this study**	3: 2 stimulated + the pool of stimulated	4: H3K27me3 in wild-type placenta	6: all	8: all	8: all except the pooled controls

**Data publication date**	Article publication: 15/01/2010 PMID: 19698761	08/01/2008	27/10/2010	30/09/2010	01/11/2010

Table 2 contains all relevant the technical information of the ChIP-on-chip and MeDIP-on-chip datasets used for the normalization approach comparison, including the dataset number as used herein, the ArrayExpress ID, the assay type, the microarray ID, species, the total number of microarrays in the dataset, the experimental content of the microarrays, a specification of the subset of microarrays used for the analyses, and the publication date of the dataset on ArrayExpress.

Sub-selections of microarrays and experimental groups were made to keep only the microarrays of sufficient quality and homogeneous replicate groups of sufficiently large size. In dataset #1, one microarray of the 17beta-estradiol stimulated group was removed because the red channel was saturated, as reported previously [26]. Instead the microarray containing a pool of stimulated samples was included. The microarrays corresponding to the untreated group were left out of the analysis. In dataset #2, only the microarrays containing the wild-type placenta H3K27me3 samples were chosen. All the microarrays from dataset #3 and #4 were used. In dataset #5 all microarrays were used, except for three containing pooled samples.

Quality control and bias assessment of the raw and normalized data was performed using the arrayQualityMetrics package [36]. Individual reports are available in additional file 1 and additional file 2 and online at http://www.bigcat.unimaas.nl/userfiles/adriaens/arrayQualityMetrics/.

### Removing technical biases through normalization

Microarray data is subject to multiple sources of variation. The goal of normalization is to remove all technical biases from the microarray data, while retaining the biological variation. There are many normalization procedures available for two-channel microarray data, but the choice for a specific procedure has to be fuelled by the characteristics of the dataset: (i) the procedure should correct all the systematic biases in the dataset diagnosed during the quality control process and (ii) the underlying assumptions of the particular method must be met. In regulation studies, there is the additional goal to retain the separation between the enriched and un-enriched components of the log-ratio distribution.

To illustrate this, data from five human and mouse ChIP-on-chip and MeDIP-on-chip datasets were normalized using six different methods: (i) LOWESS normalization [12,16] applied on each microarray individually, which assumes the log-ratio distribution is a normal distribution centered around zero; (ii) Quantile normalization [19] applied between microarrays, which equalizes the intensity distributions of all channels - green and red - together; (iii) Variance stabilizing normalization (VSN) [22], which is applied between microarrays and between channels; (iv) T-quantile normalization [19], which allows for quantile normalization of the data in subgroups and here is applied to normalize the red and green channels separately; (v) Tukey's biweight scaling, which scales the log-ratio distribution of each microarray individually using a robust Tukey's biweight estimate of the median; (vi) Peng's method [24], which performs a MA-data rotation step followed by LOWESS normalization.

NimbleGen uses Tukey's biweight scaling in-house. It consists of two steps: calculating the log-ratio between channels and subsequently correcting these by subtracting the robust Tukey's biweight estimate of the median. For this estimate, each data point is given a weight using a bi-square function. The weights assigned by this function are inversely correlated to the distance from the median, so outliers have a minimal effect on the estimate. The method developed by Peng et al. [24] makes strong assumptions regarding the shape of the MA-plot. In this approach, LOWESS normalization is preceded by a rotation step of the MA-data, which is meant to account for major dye trends. This method has been mostly applied in Drosophila [24,37,38].

### Quantifying the effect of normalization on the two-component distribution

The separation between the enriched and un-enriched components present in the data of two-channel regulation microarrays should be conserved after applying normalization. To determine this conservation, the log-ratio distribution of negative control probes (which are a measure of non-specific annealing and background fluorescence) and the log-ratio distribution of gene promoter probes were assessed before and after normalization using ROC curves. For creating the ROC curves, the negative control probes represent the negative class of outcomes, while the gene promoter probes represent the positive class of outcomes. If there are any enriched probes, the gene promoter probe distribution should extend beyond the control probe distribution in the upper quantile and is expected to have a higher mean than the control probe distribution. If this separation is retained, the ROC curves are expected to have comparable AUC values before and after normalization, while if the separation is not retained, the ROC curves will have lower AUC values after normalization.

Genomic regions known *a priori *to be enriched were used as positive controls, verifying to what extent these regions are identified after using each of the six normalization approaches. To this end, 33 well established ER-a targets [25] were chosen as positive controls for dataset #1 [26]. Enrichment of these targets was calculated using ACME with default settings and a sliding window of 750 bp [11]. For dataset #3 enrichment of the HOXA group of developmental genes was determined, which are located in a cluster on chromosome 7 and are known to be silenced and moderately to highly methylated in most tissues [27]. Enrichment p-values were calculated with ACME using default settings and a sliding window of 1000 bp. For dataset #4 the same approach was used, focussing on the Dlk1-Gtl2 cluster on chromosome 12, a region that was identified as highly methylated in the original results [28]. The other datasets lacked suitable positive controls.

Data was imported and analyzed using Bioconductor [23] in the statistical programming language R, more specifically using the ACME package [11] for enrichment finding, the limma package [39] for data normalization and the Ringo package [40] for data import and handling.

## Authors' contributions

MA and MJ carried out the analysis and interpretation of the microarray data and wrote the manuscript. CM, LE and CE helped drafting the manuscript and advised on the technical aspects and interpretation of the results. CE coordinated the project. All authors have read and approved the final manuscript.

## Supplementary Material

Additional file 1 **ArrayQualityMetrics quality control and bias assessment results (part 1)**. A ZIP file containing a folder with the results of the quality control and bias assessment generated with the arrayQualityMetrics package for datasets #1 and #2. The results are formatted as webpages. Individual results can be accessed by opening the 'index.html' file in any subfolder. An overview of all results can be accessed by combining the contents of the main folder in additional file 1 with the contents of the main folder of additional file 2, and subsequently opening the 'index.html' file in the main folder. Click here for file

Additional file 2 **ArrayQualityMetrics quality control and bias assessment results (part 2)**. A ZIP file containing a folder with the results of the quality control and bias assessment generated with the arrayQualityMetrics package for datasets #3, #4 and #5. The results are formatted as webpages. Individual results can be accessed by opening the 'index.html' file in any subfolder. An overview of all results can be accessed by combining the contents of the main folder in additional file 1 with the contents of the main folder of additional file 2, and subsequently opening the 'index.html' file in the main folder. Click here for file
