# Supplementary material for: An evaluation of two-channel ChIP-on-chip and DNA methylation microarray normalization strategies
Source: BMC Genomics. 2012 Jan 25;13:42. doi: 10.1186/1471-2164-13-42 (PMC3293711; doi:10.1186/1471-2164-13-42)
Supplement: Additional file 2 — ArrayQualityMetrics quality control and bias assessment results (part 2). A ZIP file containing a folder with the results of the quality control and bias assessment generated with the arrayQualityMetrics package for datasets #3, #4 and #5. The results are formatted as webpages. Individual results can be accessed by opening the 'index.html' file in any subfolder. An overview of all results can be accessed by combining the contents of the main folder in additional file 1 with the contents of the main folder of additional file 2, and subsequently opening the 'index.html' file in the main folder. [file 1471-2164-13-42-S2.ZIP › arrayQualityMetrics Part 2/E-GEOD-17581_VSN/index.html]

arrayQualityMetrics report for E-GEOD-17581 (VSN)


---

# arrayQualityMetrics report for E-GEOD-17581 (VSN)

---

  
- Section 1: Between array comparison

- Distances between arrays
- Principal Component Analysis

  
- Section 2: Array intensity distributions

- Boxplots
- Density plots
  
- Section 3: Variance mean dependence

- Standard deviation versus rank of the mean
  
- Section 4: Individual array quality

- MA plots
- Spatial distribution of M
- Spatial distribution of R
- Spatial distribution of G

---

### Browser compatibility

This report uses recent features of HTML 5 which have not yet been implemented by all browsers. Thus, unfortunately, browser compatibility currently needs to be considered:

- Firefox 4 - tested, works well,- Chrome 10 - tested, works well,- Safari 5 - the interactive (SVG) plots will be missing, since this browser does not support the embedding of the <svg> tag in HTML.

---

- Array metadata and outlier detection overview  

|  |  |  |  |  |  |  |  |  |  |  |  |  |  |  |
| --- | --- | --- | --- | --- | --- | --- | --- | --- | --- | --- | --- | --- | --- | --- |
|  | array | sampleNames | \*1 | \*2 | \*3 | \*4 | \*5 | \*6 | arrayNo | Species | FileNameCy3 | FileNameCy5 | Cy3 | Cy5 |
|  | 1 | GSM438047 |  |  |  |  |  |  | GSM438047 | Homo sapiens | GSM438047\_9735302\_W\_532.pair | GSM438047\_9735302\_W\_635.pair | Total | Experimental |
|  | 2 | GSM438048 |  |  |  |  |  |  | GSM438048 | Homo sapiens | GSM438048\_9736402\_532.pair | GSM438048\_9736402\_635.pair | Total | Experimental |
|  | 3 | GSM438049 |  |  |  |  |  |  | GSM438049 | Homo sapiens | GSM438049\_10059802\_532.pair | GSM438049\_10059802\_635.pair | Total | Experimental |
|  | 4 | GSM438050 |  |  |  |  |  |  | GSM438050 | Homo sapiens | GSM438050\_9735502\_532.pair | GSM438050\_9735502\_635.pair | Total | Experimental |
|  | 5 | GSM438051 |  |  |  | x | x |  | GSM438051 | Homo sapiens | GSM438051\_10059702\_532.pair | GSM438051\_10059702\_635.pair | Total | Experimental |
|  | 6 | GSM438052 | x | x |  |  |  |  | GSM438052 | Homo sapiens | GSM438052\_24281002\_532.pair | GSM438052\_24281002\_635.pair | Total | Experimental |

  
The columns named \*1, \*2, ... indicate the calls from the different outlier detection methods:

1. outlier detection by Distances between arrays
2. outlier detection by Boxplots
3. outlier detection by MA plots
4. outlier detection by Spatial distribution of M
5. outlier detection by Spatial distribution of R
6. outlier detection by Spatial distribution of G

The outlier detection criteria are explained below in the respective sections. Arrays that were called outliers by at least one criterion are marked by checkbox selection in this table, and are indicated by highlighted lines or points in some of the plots below. By clicking the checkboxes in the table, or on the corresponding points/lines in the plots, you can modify the selection. To reset the selection, reload the HTML page in your browser.

---

## Section 1: Between array comparison

- Figure 1: Distances between arrays.  

**Figure 1** (PDF file) shows a false color heatmap of the distances between arrays. The color scale is chosen to cover the range of distances encountered in the dataset. Patterns in this plot can indicate clustering of the arrays either because of intended biological or unintended experimental factors (batch effects). The distance *dab* between two arrays *a* and *b* is computed as the mean absolute difference (L1-distance) between the data of the arrays (using the data from all probes without filtering). In formula, *dab* = mean | *Mai - Mbi* |, where *Mai* is the value of the *i*-th probe on the *a*-th array. Outlier detection was performed by looking for arrays for which the sum of the distances to all other arrays, *Sa* = Σ*b* *dab* was exceptionally large. One such array was detected, and it is marked by an asterisk, \*.

+ Figure 2: Outlier detection for Distances between arrays.  

**Figure 2** (PDF file) shows a bar chart of the sum of distances to other arrays *Sa*, the outlier detection criterion from the previous figure. The bars are shown in the original order of the arrays. Based on the distribution of the values across all arrays, a threshold of 5350 was determined, which is indicated by the vertical line. One array exceeded the threshold and was considered an outlier.

- Figure 3: Principal Component Analysis.  

|  |  |  |  |  |  |  |  |  |  |  |  |  |  |  |  |  |  |
| --- | --- | --- | --- | --- | --- | --- | --- | --- | --- | --- | --- | --- | --- | --- | --- | --- | --- |
| xml version="1.0" encoding="UTF-8"? | |  |  | | --- | --- | | array |  | | sampleNames |  | | arrayNo |  | | Species |  | | FileNameCy3 |  | | FileNameCy5 |  | | Cy3 |  | | Cy5 |  | |

  
**Figure 3** (PDF file) shows a scatterplot of the arrays along the first two principal components. You can use this plot to explore if the arrays cluster, and whether this is according to an intended experimental factor (you can indicate such a factor by color using the 'intgroup' argument), or according to unintended causes such as batch effects. Move the mouse over the points to see the sample names.  
Principal component analysis is a dimension reduction and visualisation technique that is here used to project the multivariate data vector of each array into a two-dimensional plot, such that the spatial arrangement of the points in the plot reflects the overall data (dis)similarity between the arrays.  
  
  
Note: the figure is static - enhancement with interactive effects failed. This is either due to a version incompatibility of the 'SVGAnnotation' R package and your version of 'Cairo' or 'libcairo', or due to plot misformating. Please consult the Bioconductor mailing list, or contact the maintainer of 'arrayQualityMetrics' with a reproducible example in order to fix this problem.

---

## Section 2: Array intensity distributions

- Figure 4: Boxplots.  

**Figure 4** (PDF file) shows boxplots representing summaries of the signal intensity distributions of the arrays. Three panels are shown: left, red channel; middle, green channel; right, log2(ratio). Each box corresponds to one array. Typically, one expects the boxes to have similar positions and widths. If the distribution of an array is very different from the others, this may indicate an experimental problem. Outlier detection was performed on the distribution of Log2(Ratio). by computing the Kolmogorov-Smirnov statistic *Ka* between each array's distribution and the distribution of the pooled data.

+ Figure 5: Outlier detection for Boxplots.  

**Figure 5** (PDF file) shows a bar chart of the Kolmogorov-Smirnov statistic *Ka*, the outlier detection criterion from the previous figure. The bars are shown in the original order of the arrays. Based on the distribution of the values across all arrays, a threshold of 0.0662 was determined, which is indicated by the vertical line. One array exceeded the threshold and was considered an outlier.

- Figure 6: Density plots.  

|  |  |  |  |  |  |  |  |  |  |  |  |  |  |  |  |  |  |
| --- | --- | --- | --- | --- | --- | --- | --- | --- | --- | --- | --- | --- | --- | --- | --- | --- | --- |
| xml version="1.0" encoding="UTF-8"? | |  |  | | --- | --- | | array |  | | sampleNames |  | | arrayNo |  | | Species |  | | FileNameCy3 |  | | FileNameCy5 |  | | Cy3 |  | | Cy5 |  | |

  
**Figure 6** (PDF file) shows density estimates (smoothed histograms) of the data. Typically, the distributions of the arrays should have similar shapes and ranges. Arrays whose distributions are very different from the others should be considered for possible problems. Various features of the distributions can be indicative of quality related phenomena. For instance, high levels of background will shift an array's distribution to the right. Lack of signal diminishes its right right tail. A bulge at the upper end of the intensity range often indicates signal saturation.

---

## Section 3: Variance mean dependence

- Figure 7: Standard deviation versus rank of the mean.  

**Figure 7** (PDF file) shows a density plot of the standard deviation of the intensities across arrays on the *y*-axis versus the rank of their mean on the *x*-axis. The red dots, connected by lines, show the running median of the standard deviation. After normalisation and transformation to a logarithm(-like) scale, one typically expects the red line to be approximately horizontal, that is, show no substantial trend. In some cases, a hump on the right hand of the x-axis can be observed and is symptomatic of a saturation of the intensities.

---

## Section 4: Individual array quality

- Figure 8: MA plots.  

**Figure 8** (PDF file) shows MA plots. M and A are defined as:  
M = log2(I1) - log2(I2)  
A = 1/2 (log2(I1)+log2(I2)),  
where I1 and I2 are the intensities of the two channels. Typically, we expect the mass of the distribution in an MA plot to be concentrated along the M = 0 axis, and there should be no trend in M as a function of A. If there is a trend in the lower range of A, this often indicates that the arrays have different background intensities; this may be addressed by background correction. A trend in the upper range of A can indicate saturation of the measurements; in mild cases, this may be addressed by non-linear normalisation (e.g. quantile normalisation).  
Outlier detection was performed by computing Hoeffding's statistic *Da* on the joint distribution of A and M for each array. The value of *Da* is shown in the panel headings. 0 arrays had *Da*>0.15 and were marked as outliers. For more information on Hoeffing's *D*-statistic, please see the manual page of the function hoeffd in the Hmisc package.

+ Figure 9: Outlier detection for MA plots.  

**Figure 9** (PDF file) shows a bar chart of the Hoeffding's statistic *Da*, the outlier detection criterion from the previous figure. The bars are shown in the original order of the arrays. A threshold of 0.15 was used, which is indicated by the vertical line. None of the arrays exceeded the threshold and was considered an outlier.

- Figure 10: Spatial distribution of M.  

**Figure 10** (PDF file) shows false color representations of the arrays' spatial distributions of feature intensities (M). Normally, when the features are distributed randomly on the arrays, one expects to see a uniform distribution; control features with particularly high or low intensities may stand out. The color scale is proportional to the ranks of the probe intensities. Note that the rank scale has the potential to amplify patterns that are small in amplitude but systematic within an array. It is possible to switch off the rank scaling by modifying the argument scale in the call of the aqm.spatial function.  
Outlier detection was performed by computing *Fa* , the sum of the absolutes value of low frequency Fourier coefficients, as a measure of large scale spatial structures. The value of *Fa* is shown in the panel headings.

+ Figure 11: Outlier detection for Spatial distribution of M.  

**Figure 11** (PDF file) shows a bar chart of the *Fa*, the outlier detection criterion from the previous figure. The bars are shown in the original order of the arrays. Based on the distribution of the values across all arrays, a threshold of 0.0276 was determined, which is indicated by the vertical line. One array exceeded the threshold and was considered an outlier.

- Figure 12: Spatial distribution of R.  

**Figure 12** (PDF file) shows false color representations of the arrays' spatial distributions of feature intensities (R). Normally, when the features are distributed randomly on the arrays, one expects to see a uniform distribution; control features with particularly high or low intensities may stand out. The color scale is proportional to the ranks of the probe intensities. Note that the rank scale has the potential to amplify patterns that are small in amplitude but systematic within an array. It is possible to switch off the rank scaling by modifying the argument scale in the call of the aqm.spatial function.  
Outlier detection was performed by computing *Fa* , the sum of the absolutes value of low frequency Fourier coefficients, as a measure of large scale spatial structures. The value of *Fa* is shown in the panel headings.

+ Figure 13: Outlier detection for Spatial distribution of R.  

**Figure 13** (PDF file) shows a bar chart of the *Fa*, the outlier detection criterion from the previous figure. The bars are shown in the original order of the arrays. Based on the distribution of the values across all arrays, a threshold of 0.0238 was determined, which is indicated by the vertical line. One array exceeded the threshold and was considered an outlier.

- Figure 14: Spatial distribution of G.  

**Figure 14** (PDF file) shows false color representations of the arrays' spatial distributions of feature intensities (G). Normally, when the features are distributed randomly on the arrays, one expects to see a uniform distribution; control features with particularly high or low intensities may stand out. The color scale is proportional to the ranks of the probe intensities. Note that the rank scale has the potential to amplify patterns that are small in amplitude but systematic within an array. It is possible to switch off the rank scaling by modifying the argument scale in the call of the aqm.spatial function.  
Outlier detection was performed by computing *Fa* , the sum of the absolutes value of low frequency Fourier coefficients, as a measure of large scale spatial structures. The value of *Fa* is shown in the panel headings.

+ Figure 15: Outlier detection for Spatial distribution of G.  

**Figure 15** (PDF file) shows a bar chart of the *Fa*, the outlier detection criterion from the previous figure. The bars are shown in the original order of the arrays. Based on the distribution of the values across all arrays, a threshold of 0.0435 was determined, which is indicated by the vertical line. None of the arrays exceeded the threshold and was considered an outlier.

---

This report has been created with arrayQualityMetrics 3.10.0 under R version 2.14.0 (2011-10-31).

---

  
