# Supplementary material for: An evaluation of two-channel ChIP-on-chip and DNA methylation microarray normalization strategies
Source: BMC Genomics. 2012 Jan 25;13:42. doi: 10.1186/1471-2164-13-42 (PMC3293711; doi:10.1186/1471-2164-13-42)
Supplement: Additional file 1 — ArrayQualityMetrics quality control and bias assessment results (part 1). A ZIP file containing a folder with the results of the quality control and bias assessment generated with the arrayQualityMetrics package for datasets #1 and #2. The results are formatted as webpages. Individual results can be accessed by opening the 'index.html' file in any subfolder. An overview of all results can be accessed by combining the contents of the main folder in additional file 1 with the contents of the main folder of additional file 2, and subsequently opening the 'index.html' file in the main folder. [file 1471-2164-13-42-S1.ZIP › arrayQualityMetrics Part 1/index.html]

arrayQualityMetrics reports


---

# arrayQualityMetrics reports for

## "An evaluation of two-channel ChIP-on-chip and DNA methylation microarray normalization strategies"

|  |  |  |  |  |  |  |  |  |  |  |  |  |  |  |  |  |  |  |  |  |  |  |  |  |  |  |  |  |  |  |  |  |  |  |  |  |  |  |  |  |  |  |  |  |  |  |  |  |  |  |  |  |  |
| --- | --- | --- | --- | --- | --- | --- | --- | --- | --- | --- | --- | --- | --- | --- | --- | --- | --- | --- | --- | --- | --- | --- | --- | --- | --- | --- | --- | --- | --- | --- | --- | --- | --- | --- | --- | --- | --- | --- | --- | --- | --- | --- | --- | --- | --- | --- | --- | --- | --- | --- | --- | --- | --- |
| Dataset number | Dataset name | Raw data | Loess | Quantile | VSN | Peng | NimbleGen | T-quantile || 1 | ER-alpha | raw data | norm Loess | norm Quantile | norm VSN | norm Peng | norm Nimblegen | norm T-quantile |
| 2 | E-TABM-529 | raw data | norm Loess | norm Quantile | norm VSN | norm Peng | norm Nimblegen | norm T-quantile |
| 3 | E-GEOD-17581 | raw data | norm Loess | norm Quantile | norm VSN | norm Peng | norm Nimblegen | norm T-quantile |
| 4 | E-GEOD-24286 | raw data | norm Loess | norm Quantile | norm VSN | norm Peng | norm Nimblegen | norm T-quantile |
| 5 | E-GEOD-22831 | raw data | norm Loess | norm Quantile | norm VSN | norm Peng | norm Nimblegen | norm T-quantile |

Outliers found on raw data for six quality metrics:
  

|  |  |  |  |  |  |  |  |  |  |  |  |  |  |  |  |  |  |  |  |  |  |  |  |  |  |  |  |  |  |  |  |
| --- | --- | --- | --- | --- | --- | --- | --- | --- | --- | --- | --- | --- | --- | --- | --- | --- | --- | --- | --- | --- | --- | --- | --- | --- | --- | --- | --- | --- | --- | --- | --- |
| Dataset | ER-alpha | | | E-TABM-529 | | | | E-GEOD-17581 | | | | | | | | E-GEOD-24286 | | | | | | | | E-GEOD-22831 | | | | | | | |
| Array number | 1 | 2 | 3 | 1 | 2 | 3 | 4 | 1 | 2 | 3 | 4 | 5 | 6 | 7 | 8 | 1 | 2 | 3 | 4 | 5 | 6 | 7 | 8 | 1 | 2 | 3 | 4 | 5 | 6 | 7 | 8 |
| Distances between arrays |  |  |  |  |  |  |  |  |  |  |  |  |  |  |  |  |  |  |  |  |  |  |  |  |  |  |  |  |  |  |  |
| Boxplots |  |  |  |  |  |  |  | x |  |  |  |  |  |  |  |  |  |  |  |  |  |  |  | x |  |  |  |  |  |  |  |
| MA plots |  |  |  |  | x | x | x |  |  |  |  |  |  |  |  |  |  |  |  |  |  | x |  |  |  |  |  |  |  |  |  |
| Spatial distribution of M |  |  |  |  |  |  |  |  |  |  |  |  |  |  |  |  |  |  |  |  |  |  |  |  |  |  |  |  |  |  |  |
| Spatial distribution of R |  |  |  |  |  |  |  |  |  |  |  | x |  |  |  |  |  |  |  |  |  |  |  |  |  |  |  |  |  |  |  |
| Spatial distribution of G |  |  |  |  |  |  |  |  |  |  |  |  |  |  |  |  |  |  |  |  |  |  |  |  |  |  |  |  |  |  |  |
